# Emotion-Related Consciousness Detection in Patients With Disorders of Consciousness Through an EEG-Based BCI System

**DOI:** 10.3389/fnhum.2018.00198

**Published:** 2018-05-15

**Authors:** Jiahui Pan, Qiuyou Xie, Haiyun Huang, Yanbin He, Yuping Sun, Ronghao Yu, Yuanqing Li

**Affiliations:** ^1^School of Software, South China Normal University, Guangzhou, China; ^2^Centre for Hyperbaric Oxygen and Neurorehabilitation, General Hospital of Guangzhou Military Command, Guangzhou, China; ^3^School of Automation Science and Engineering, South China University of Technology, Guangzhou, China; ^4^Guangzhou Key Laboratory of Brain Computer Interaction and Applications, Guangzhou, China

**Keywords:** emotion recognition, disorders of consciousness (DOC), brain computer interface (BCI), P300, consciousness detection

## Abstract

For patients with disorders of consciousness (DOC), such as vegetative state (VS) and minimally conscious state (MCS), detecting and assessing the residual cognitive functions of the brain remain challenging. Emotion-related cognitive functions are difficult to detect in patients with DOC using motor response-based clinical assessment scales such as the Coma Recovery Scale-Revised (CRS-R) because DOC patients have motor impairments and are unable to provide sufficient motor responses for emotion-related communication. In this study, we proposed an EEG-based brain-computer interface (BCI) system for emotion recognition in patients with DOC. Eight patients with DOC (5 VS and 3 MCS) and eight healthy controls participated in the BCI-based experiment. During the experiment, two movie clips flashed (appearing and disappearing) eight times with a random interstimulus interval between flashes to evoke P300 potentials. The subjects were instructed to focus on the crying or laughing movie clip and to count the flashes of the corresponding movie clip cued by instruction. The BCI system performed online P300 detection to determine which movie clip the patients responsed to and presented the result as feedback. Three of the eight patients and all eight healthy controls achieved online accuracies based on P300 detection that were significantly greater than chance level. P300 potentials were observed in the EEG signals from the three patients. These results indicated the three patients had abilities of emotion recognition and command following. Through spectral analysis, common spatial pattern (CSP) and differential entropy (DE) features in the delta, theta, alpha, beta, and gamma frequency bands were employed to classify the EEG signals during the crying and laughing movie clips. Two patients and all eight healthy controls achieved offline accuracies significantly greater than chance levels in the spectral analysis. Furthermore, stable topographic distribution patterns of CSP and DE features were observed in both the healthy subjects and these two patients. Our results suggest that cognitive experiments may be conducted using BCI systems in patients with DOC despite the inability of such patients to provide sufficient behavioral responses.

## 1. Introduction

Patients with severe brain injury may suffer from disorders of consciousness (DOC), including coma, vegetative state (VS) and minimally conscious state (MCS). Keystones in diagnosing these disorders are the acquisition of voluntary responses, such as command following and functional communication, which indicate emergence from VS and MCS, respectively (Noirhomme et al., 2013). Currently, the clinical diagnosis of DOC patients is generally based on behavioral scales, such as the Coma Recovery Scale-Revised (CRS-R), which rely on overt motor responses to external stimuli at the time of observation (Seel et al., 2010). However, motor responses may be difficult to discern or inconsistent in this patient group, and it is becoming increasingly clear that relying on an overt behavioral response may result in misdiagnosis of a patients level of consciousness (Cruse and Owen, 2010; Coyle et al., 2017). In recent years, researchers have employed electroencephalography (EEG) and functional magnetic resonance imaging (fMRI) techniques (Owen et al., 2002; Laureys et al., 2004; Di et al., 2007; Monti et al., 2010; Cruse et al., 2012a; Li et al., 2015b; Wang et al., 2017) to detect residual brain functions and provide motor-independent evidence of consciousness in certain patients with DOC (see Noirhomme et al., 2013; Kotchoubey, 2017; Lancioni et al., 2017 for reviews).

Emotion recognition is an important brain function associated with many cognitive functions, including selective attention, working memory, language abilities, and decision making (Kohler et al., 2000; Molina et al., 2009). Several neuroimaging and electrophysiological studies have proposed probing the neural machanism of emotion recognition. For instance, fMRI findings have suggested that the amygdala and the orbitofrontal cortex are key areas in the brains emotion recognition system. Two important mechanisms for emotion recognition are constructing a simulation of the emotion observed in the perceiver and modulating sensory cortices through top-down influences (Adolphs, 2002). Many studies have also reported that injury sites in neurological patients could result in deficits in emotion recognition. For instance, some patients who suffer from stroke have shown difficulties in emotion recognition, that are more frequently observed in individuals with right brain damage than in those with left brain damage (Yuvaraj et al., 2013). Several studies have also reported emotion recognition deficits in patients with schizophrenia (Kohler et al., 2000; Taylor and Iii, 2012; Kayser et al., 2014; Corcoran et al., 2015; Bilgi et al., 2017), and the results have suggested that impairments in auditory, olfactory, or visual function may lead to deficits in emotion recognition. Furthermore, emotion recognition tasks have been included in the Functional Emotional Assessment Scale (FEAS), the Development Neuropsychological Assessment-II (NEPSY-II) and the Montreal Cognitive Assessment (MoCA), which are commonly used to evaluate cognitive impairments in patients with schizophrenia, attention deficit hyperactivity disorder (ADHD), and Parkinsons disease (Solomon et al., 2007; Marneweck and Hammond, 2014; Pitzianti et al., 2017). However, emotion recognition tasks do not include in the clinical behavioral scales such as the CRS-R for patients with DOC. Thus far, whether patients with DOC can recognize emotion remains unknown. One possible reason is that these DOC patients who are severely lack of motor ability cannot provide sufficient motor responses for emotion recognition-based behavioral experiments. By exploring emotion recognition in patients with DOC, we may be able to more thoroughly evaluate residual cognitive functions and determine the extent to which the multiple brain functions associated with emotion recognition are impaired after severe brain injury.

Brain-computer interfaces (BCIs) allow non-muscular communication and control by directly translating brain activities into computer control signals, thereby enabling users with motor disabilities to convey their intent to the external world (McFarland and Wolpaw, 2004). Therefore, BCIs may allow the exploration of residual cognition functions, such as emotion recognition, in patients with DOC. Recently, several BCI paradigms have been proposed for patients with DOC (Coyle et al., 2012; Lulé et al., 2012; Müller-Putz et al., 2012, 2013; Gibson et al., 2016; Wang et al., 2017). Lulé et al. (2012) used a 4-choice auditory P300-based BCI system to detect consciousness in 13 MCS, 3 VS and 2 locked-in syndrome (LIS) patients. Among the 18 DOC patients, one LIS patient presented significant accuracy 60%. Coyle and his colleagues (Coyle et al., 2015) developed a motor imagery-based BCI with auditory or visual feedback to detect consciousness in 4 MCS patients. The results indicated that all four patients had the capacity to use a simple BCI system with a peak mean classification accuracy above 70%. In our previous study (Pan et al., 2014), a visual hybrid P300 and steady-state visual evoked potentials (SSVEPs) BCI was developed to detect consciousness in eight patients with DOC (4 VS, 3 MCS, and 1 LIS), and three of them (1 VS, 1 MCS, and 1 LIS) achieved BCI accuracies significantly higher than the chance level. However, BCI-based consciousness detection systems for use by patients with DOC remain in their infancy. The performance of BCIs designed for DOC patients is generally poor due to their limited cognitive levels. Furthermore, because patients with DOC have suffered from severe brain injuries, large differences in EEG signals exist between these patients and healthy individuals. Thus, researchers strive to develop novel BCIs to improve the performance of consciousness detection.

Recent studies have validated that the accuracy and speed of BCIs can be improved by the emotion elicitation techniques and emotion-related processing (see Molina et al., 2009 for review). For instance, it has been shown that stimuli containing an affective component elicit latency and amplitude differences in the characteristic peaks of event-related potentials (ERPs), which can enhance the electrophysiological sources of control used in BCI systems (Kayser et al., 2000). To date, mirror neuron system (MNS)-based emotion elicitation techniques have been widely used. According to MNS mechanism, simple observation of emotional facial expressions done by another individuals might evoked the same brain activity as if they experienced the corresponding emotion themselves (Petrantonakis and Hadjileontiadis, 2011). Furthermore, many studies have focused on the EEG-based emotion recognition in healthy individuals. The power spectra of EEGs have also been assessed in different frequency bands to examine their relationships with emotional states (Wang et al., 2014). Previous studies have reported several spectral power changes in various brain regions that have been associated with emotional responses; these changes have included theta (4–7 Hz) power changes at the right parietal lobe (Aftanas et al., 2004), alpha (8–13 Hz) power asymmetry at anterior areas of the brain (Allen et al., 2004), beta (14–30 Hz) power asymmetry at the parietal region (Schutter et al., 2001), and gamma (31–50 Hz) power changes at the right parietal regions (Li and Lu, 2009). Lin et al. reported an offline accuracy of 82.29% by using spectral power asymmetries across multiple frequency bands as features and a support vector machine (SVM) classifier to characterize EEG signals into four emotional states during music listening (Lin et al., 2010). Lv and colleagues investigated stable EEG patterns for emotion recognition using a graph regularized extreme learning machine (GELM) (Zheng et al., 2016). They found that the GELM with differential entropy (DE) features obtained average classification accuracies of 69.67% for the DEAP dataset and 91.07% for their SEED dataset based on 5-fold cross-validations. In our previous study (Pan et al., 2016), we employed facial expression pictures to develop an EEG-based BCI system for emotion recognition of happiness and sadness, and we achieved an average online accuracy of 87.5% using an SVM with common spatial pattern (CSP) features. To the best of our knowledge, emotion recognition, which is expected to be impaired to a certain degree, has not been studied in patients with DOC.

Considering the abovementioned factors, we proposed an EEG-based BCI system for the detection of consciousness in patients with DOC. Eight patients with DOC (3 VS and 5 MCS patients) and eight healthy controls participated in the BCI-based experiment. During the experiment, two movie clips flashed (appearing and disappearing) eight times with a random interstimulus interval to evoke P300 potentials. As the movie clip flashed, the corresponding spoken sound was simultaneously played. The subjects were instructed to focus on the crying or laughing movie clip and to count the flashes of the corresponding movie clip cued by the delivered instruction. The BCI system performed online P300 detection to determine which movie clip the patients attended to and presented the result as feedback. Three of the eight patients achieved online accuracies based on P300 detection that were significantly greater than chance level. Emotion recognition and the ability to follow commands were demonstrated in these three patients. Through the spectral analysis, CSP and DE features in the delta, theta, alpha, beta, and gamma frequency bands were employed to classify the EEG signals during the crying and laughing movie clips. Two patients and all eight healthy controls achieved offline accuracies significantly greater than chance level in the offline spectral analysis. Additionally, stable topographic distribution patterns of CSP and DE features were observed in both the healthy subjects and these two patients. Our results suggest that cognitive experiments may be conducted using BCI systems in patients with DOC despite the inability of such patients to provide sufficient behavioral responses.

## 2. Methods

### 2.1. Subjects

Eight severely brain-damaged patients (four males; three with VS and five with MCS; mean age ± SD, 33.3 ± 14.8 years old; see Table 1) from a local hospital participated in this experiment. All of the patients included in this study were recruited according to predefined inclusion criteria. Inclusion criteria were as follows: (a) no centrally acting drugs; (b) no sedation within the prior 48 h; (c) periods of eye opening; (d) no history of impaired visual or auditory acuity before brain injury; and (e) diagnosis of VS or MCS after a traumatic brain injury (TBI), anoxic brain injury, or cerebrovascular accident. This study was approved by the Ethical Committee of the General Hospital of Guangzhou Military Command of the Peoples Liberation Army in Guangzhou, and complies with the Code of Ethics of the World Medical Association (Declaration of Helsinki). These patients or their legal surrogates provided written informed consent for participation in the BCI experiments and publication of their individual details in this manuscript.

**Table 1:** Summary of patients' clinical status.

**Patient**	**Age**	**Gender**	**Clinical diagnosis**	**Etiology**	**Time since injury (months)**	**CRS-R score (subscores)**	
						**Before experiment**	**After 1 month**
P1	33	F	VS	NTBI	2	7 (1-1-2-1-0-2)	7 (1-1-2-1-0-2)
P2	49	F	MCS	NTBI	6	9 (1-3-2-1-0-2)	11 (3-3-2-1-0-2)
P3	26	M	VS	TBI	2	5 (1-1-1-0-0-2)	13 (3-3-3-1-1-2)
P4	23	M	MCS	TBI	3	10 (2-3-2-1-0-2)	10 (2-3-2-1-0-2)
P5	37	M	MCS	TBI	2	9 (1-3-2-1-0-2)	9 (1-3-2-1-0-2)
P6	18	F	MCS	TBI	2	8 (1-1-3-1-0-2)	8 (1-1-3-1-0-2)
P7	60	F	VS	TBI	4	7 (1-1-2-1-0-2)	7 (1-1-2-1-0-2)
P8	20	M	MCS	TBI	2	8 (1-1-3-1-0-2)	8 (1-1-3-1-0-2)

*CRS-R, coma recovery scale-revised; NTBI, non-traumatic brain injury; and TBI, traumatic brain injury; CRS-R subscales: Auditory, visual, motor, oromotor, communication, and arousal functions*.

The diagnoses of VS or MCS were based on the CRS-R, which contains 23 items organized in 6 subscales addressing auditory, visual, motor, oromotor, communication, and arousal processes. Scoring on each subscale is based on the specific operational criteria. The eight patients attended two CRS-R assessments: one during the week before the experiment and another at 1 month after the experiment. The CRS-R scores for each patient are presented in Table 1. Additionally, eight healthy subjects (HC1, HC2, HC3, HC4, HC5, HC6, HC7, and HC8) (seven males; mean age ± SD, 29.2 ± 3.3 years old) participated in the experiment as a control group.

### 2.2. Stimuli and graphic user interface (GUI)

Movie clips of emotional facial expression were used as stimuli. Eighty movie clips including video and audio recordings of 1,400 ms in duration, which were used in our previous study (Li et al., 2015a), were selected as stimuli. These audiovisual stimuli consisted of two sets of emotional movie clips: 40 laughing movie clips and 40 crying movie clips, corresponding to happy and sad emotional states, respectively. All the movie clips were edited using Premiere Pro software, version CS6 (Adobe, San Jose, CA, USA) to ensure identical overall luminance levels on a gray scale. In addition, they were edited using Adobe Audition software, version CS6 (Adobe, San Jose, CA, USA) to ensure that the audio recordings had identical power levels. Note that after the experiments, each healthy subject was asked to rate the emotional content of each movie clip using the self-assessment manikin (SAM, Bradley and Lang, 1994). The SAM evaluation rating of the valence-arousal scales were (7.09 ± 0.91, 5.21 ± 1.67) and (2.41 ± 0.81, 4.27 ± 1.21) for laughing and crying movie clips, respectively.

**Figure 1:**
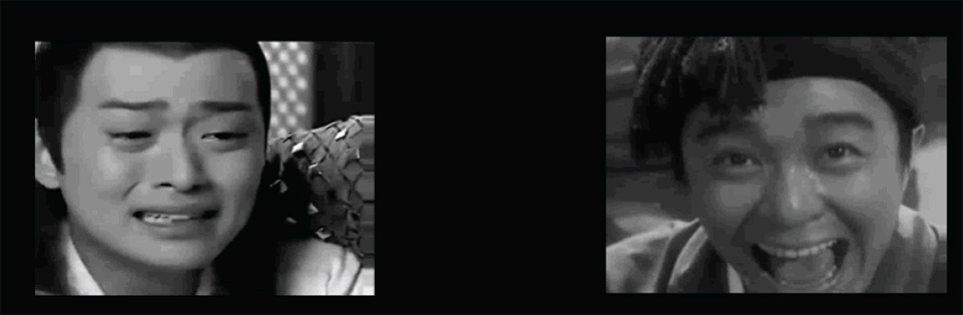
GUI of the BCI, in which a crying movie clip and a laughing movie clip are arranged on the left and right sides, respectively. The two movie clips flashed (appearing and disappearing) on the black background with a random inter-stimulus interval.

The GUI used in this study is illustrated in Figure 1. Two movie clips, a crying movie clip and a laughing movie clip, were pseudorandomly chosen from the two sets of movie clips and were displayed on the left and right sides of the GUI. The size (area) of each movie clip was 9 cm × 7.2 cm, and the horizontal distance between the two movie clips in the GUI was 6 cm. The ratio of the movie clip size to the GUI size was set at 0.12:1. Two loudspeakers were placed behind the monitor to present the auditory stimuli. The two videos clips flashed (appearing and disappearing), with each clip appearing for 1,400 ms. When the video clip appeared, the corresponding audio clip was simultaneously played from the speaker. Specifically, one movie clip (e.g., the crying movie clip in Figure 1) was flashed eight times, and then the other movie clip (e.g., the laughing movie clip in Figure 1) was flashed eight times. The interval between two consecutive movie clips was randomly chosen from among 500, 600, 700, 800, 900, 1,000, 1,100, and 1,200 ms. The patients were instructed to focus on one movie clip (e.g., the crying or laughing movie clip) and to count the flashes of the corresponding movie clip.

### 2.3. Data acquisition

A NuAmps amplifier (Neuroscan Compumedics, USA) and an EEG cap (LT 37) were used to record 30-electrode scalp EEG signals for data acquisition. The EEG signals were amplified, sampled at 250 Hz, bandpass filtered between 0.1 and 60 Hz, and referenced to the right mastoid. The impedances of all electrodes were kept below 5*kΩ*. In order to remove ocular movement artifacts from the EEG signal, an electrooculogram (EOG) was captured from two pairs of electrodes (“HEOR” and “HEOL”;“VEOU” and “VEOL”).

### 2.4. Experimental procedures

During the experiment, patients were seated on a comfortable wheelchair and repeatedly instructed to avoid blinking or moving their body. Before the experiment, preliminary screening was conducted to explain the procedure to patients.

In this experiment, two experimental runs were conducted: one for calibration and the other for online evaluation. Each subject first performed a calibration run of 20 trials with the GUI in Figure 1 to collect training data. In this study, we collected a small training dataset for each subject, because the BCI system was designed mainly for patients with DOC who are easily fatigued during experiments. We trained the initial SVM classifier using the EEG data from the calibration run. Each subject subsequently performed an evaluation run of 50 trials.

The online evaluation run contained five blocks, each of which was composed of 10 trials and was conducted on separate days because the patients were easily fatigued. The experimental procedure of one trial in this experiment is illustrated as follows. Two pairs of audiovisual stimuli were first constructed, for which one pair of audiovisual stimuli corresponded to a laughing movie clip and the other pair corresponded to a crying movie clip. These audiovisual stimuli were pseudorandomly chosen from two sets of emotional movie clips, which consisted of 40 laughing movie clips and 40 crying movie clips corresponding to happy and sad emotional states, respectively. Each trial began by presenting audiovisual instructions, which lasted 10 s. The subject was instructed to “Pay attention to the happy/sad movie clips and to count the flashes of the happy/sad movie clips.” Note that the two emotional states appeared in a pseudo-random order, with half the trials containing the happy movie clip and the other half containing the sad movie clip. Following presentation of the instructions, the two pairs of audiovisual stimuli, constructed as described above, were presented. The two video clips flashed (appearing and disappearing), with each appearing for 1,400 ms. When the video clip appeared, the corresponding audio clip was simultaneously played from the speakers. The interval between two consecutive audiovisual stimuli was randomly chosen from among 500, 600, 700, 800, 900, 1,000, 1,100, and 1,200 ms. After the 36 s audiovisual presentation, the BCI algorithm determined the target movie clip. If the detection result was correct, positive feedback consisting of the laughing or crying facial expression in the given trial and auditory applause was delivered for 4 s; otherwise, no feedback was given. Before the next trial beginning, there was a break of at least 10 s depending on the patient's level of fatigue. If the patient showed continuous body movements (e.g., coughing) or decreased arousal (i.e., closed eyes for a period of 5 s) in a trial, the trial was rejected to reduce artifacts, and the next trial began after the patient re-awakened and re-stabilized.

### 2.5. Data processing and algorithm

The P300 detection algorithms and spectral analysis methods were designed separately. The former was used to detect whether the subject responsed to the target movie clip in real-time, and the latter was used to detect whether the EEG signals during the crying and laughing movie clips could be classified using emotion-related CSP and DE features. The algorithms and analysis methods used in this study are described in the following sections.

#### 2.5.1. Online P300 detection

First, the EEG signals were filtered between 0.1 and 10 Hz. Then, we extracted a segment of the EEG signal from each channel (0–600 ms from the start of the movie clip) for each flash of a movie clip. This segment was down-sampled by a factor of 5 to obtain a data vector (with a length of 30) from each channel. Next, we concatenated the vectors from all channels to obtain a new data vector (with a length of 900) corresponding to the movie clip flashes. In order to improve signal-to-noise ratio, we constructed a feature vector corresponding to each movie clip by averaging the data vectors across the eight flashes in a trial. Finally, we applied the SVM classifier to the two feature vectors corresponding to the two types of movie clips, and two SVM scores were obtained for each trial. Specifically, the SVM classifier was first trained using the training data, in which the feature vectors corresponding to the target and non-target movie clips were labeled +1 and -1, respectively. For each test trial, the trained SVM was applied to the two feature vectors corresponding to the two movie clips, and the predicted target movie clip was the movie clip corresponding to the higher score.

#### 2.5.2. Offline spectral analysis

In the offline analysis, we performed spectral analysis by stimulating the online training and testing. The EEG data were first bandpass filtered over the five frequency bands: delta (1–3 Hz), theta (4–7 Hz), alpha (8–13 Hz), beta (14–30 Hz), and gamma (31–50 Hz). After bandpass filtering, we extracted two segments (one for the laughing movie clip and one for the crying movie clip) of EEG data over an 18 s period (4,500 data points) for each channel and each frequency band. For each segment, we extracted the CSP feature and the DE feature, as described below.

First, a CSP spatial filter, **W**, was learned in a subject-specific manner to enhance the separability between the two emotion classes (i.e., happiness and sadness) in the training data. Specifically, a training dataset was collected for each subject during a calibration run, in which 20 trials of the instructed emotion recognition task were performed. The spatial filter matrix **W** was constructed by the well-known joint diagonalization method (Blanchard and Blankertz, 2004). For each trial, the CSP features were then extracted using the following filter:

$$\begin{array}{l} \text{fm}=\log_{10}\left(diag\left(\bar{\text{W}}\text{E}{\text{E}}^{T}{\bar{\text{W}}}^{T}\right)\right) \end{array}$$

where **fm** denotes the CSP feature vector, **W** is a submatrix composed of the first and last three rows of **W**, and **E** is an EEG data matrix corresponding to one trial. In Eq. (1), *diag*(**WEE**^*T*^**W**^*T*^) is a vector composed of all entries on the diagonal line of the matrix **WEE**^*T*^**W**^*T*^, and the operator log_10_(.) is used to calculate the logarithm of each entry of the vector. In this study, we selected the top and bottom three components from **W** that best separated the two emotion classes. Furthermore, their logarithm variances were calculated, and a 6-D CSP feature vector was constructed for each frequency band.

We then used the discrete Fourier transform to calculate the power spectral density for each segment. The DE was defined as the logarithmic power spectral density for a fixed-length EEG sequence. For each frequency band, we constructed the DE feature vector by concatenating the differential entropies from all of the channels.

Two feature vectors of a trial corresponding to the two segments (one for the laughing movie clip and one for the crying movie clip) were then obtained by concatenating all of the CSP and DE feature vectors of all frequency bands. An SVM classifier was first trained using the training data, in which the feature vectors corresponding to the laughing and crying movie clips were labeled +1 and -1, respectively. For a test EEG trial, two feature vectors were first obtained, as described above, and then were fed into the SVM classifier to obtain two SVM scores. If both the SVM score corresponding to the laughing movie clip and the sum of the two SVM scores were positive, the predicted emotional state is happiness. If both the SVM score corresponding to the crying movie clip and the sum of the two SVM scores are negative, the predicted emotional state was sadness.

#### 2.5.3. Performance measures

For each subject, the ratio of trials with correct responses (hits) to the total number of trials was calculated as the accuracy rate. To obtain the significance level of the accuracy, the χ^2^ statistical test was performed. Specifically, the χ^2^ statistic was calculated as follows (Kübler and Birbaumer, 2008; Pan et al., 2014):

(1) $$\begin{array}{r} \chi^{2}=\overset{k}{\underset{i\;=\;1}{\sum }}\frac{{\left(fo_{i}-fe_{i}\right)}^{2}}{fe_{i}} \end{array}$$

where *fo*_*i*_ and *fe*_*i*_ were the observed and expected frequencies of the *i*th class (*i* = 1, 2, …, *k*) (degree of freedom: 1). In this study, the observations fell into two classes (hit and miss). Therefore, the chance level that the target was selected was 0.5, whereas the chance level for selecting a non-target was 0.5. Considering that 50 trials of the BCI evaluation were conducted for each subject, the expected *fo*_1_ and *fo*_2_ were 25 and 25, respectively. Specifically, *fo*_1_ and *fo*_2_ was the number of times that the target (i=1) or a non-target (i=2) was determined in the BCI evaluation. We obtained a value of 3.84 for χ^2^ test with a significance level of *p* = 0.05, corresponding to 32 hits in 50 trials or an accuracy of 64%.

## 3. Results

Table 2 summarizes the accuracy rates of the P300 detection (online) and the spectral analysis (offline) for the experiment. For the P300 detection, three of the eight patients (patients P2, P3, and P6) achieved the significant accuracies (66–78%), corresponding to a significance level of 0.05. For patients P1, P4, P5, P7, and P8, the accuracies of the P300 detection were not significant. For spectral analysis, two of the eight patients (patients P2 and P3) achieved accuracies ranging from 66 to 68%, whereas patients P1, P4, P5, P6, P7, and P8 achieved accuracies lower than 64% (ranging from 48 to 60%). Furthermore, all eight healthy subjects (HC1, HC2, HC3, HC4, HC5, HC6, HC7, and HC8) achieved the significant accuracies (ranging from 78 to 100%) for both P300 detection and spectral analysis.

**Table 2:** Accuracy rates of the P300 detection (online) and the spectral analysis (offline) for the subjects.

**Subjects**	**Online accuracy (P300 detection)**	***p*-value**	**Offline accuracy (spectral analysis)**	***p*-value**
P1	52	0.778	52	0.778
P2	**78**	<0.001	**68**	0.011
P3	**68**	0.011	**66**	0.047
P4	54	0.572	50	1.000
P5	56	0.396	48	0.778
P6	**66**	0.047	60	0.157
P7	50	1.000	54	0.572
P8	58	0.258	58	0.258
HC1	**100**	<0.001	**90**	<0.001
HC2	**100**	<0.001	**76**	<0.001
HC3	**96**	<0.001	**90**	<0.001
HC4	**100**	<0.001	**76**	<0.001
HC5	**94**	<0.001	**70**	0.005
HC6	**78**	<0.001	**68**	0.011
HC7	**96**	<0.001	**76**	<0.001
HC8	**98**	<0.001	**82**	<0.001

*The accuracies significantly higher than the chance level 50% (accuracy ≥64% or p ≤ 0.05) are highlighted in bold*.

**Figure 2:**
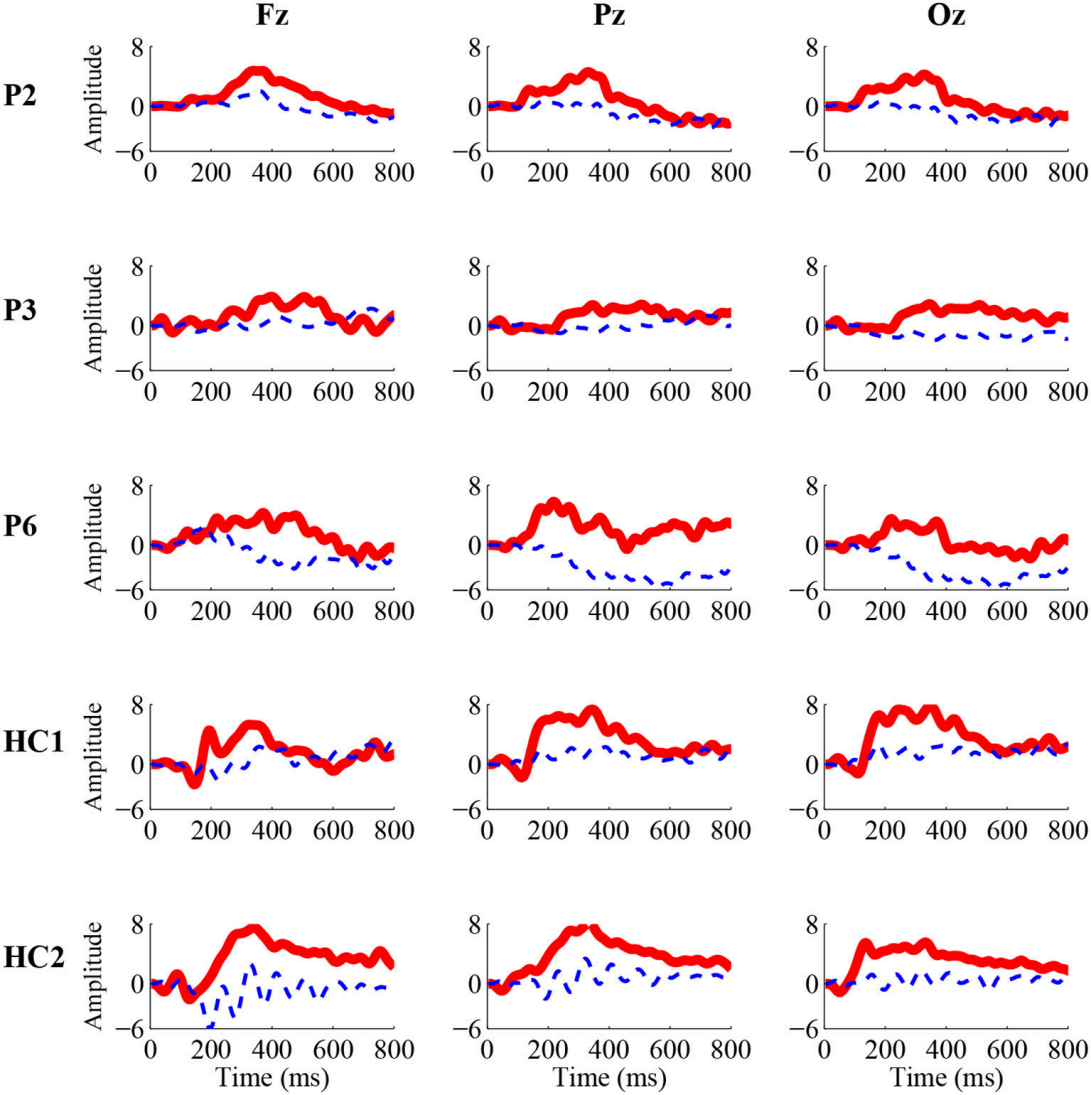
Grand-average P300 ERP waveforms from the “Fz” (left), “Cz” (middle), and “Pz” (right) electrodes in the online experiment for three patients (P2, P3, and P6) and two healthy controls (HC1 and HC2). The solid red curves containing the P300 component correspond to the target movie clip, while the dashed blue curves without the P300 component correspond to the non-target movie clip.

For the three patients (P2, P3, and P6) and two healthy controls (HC1 and HC2) whose accuracies of the P300 detection were significant, we calculated ERP waveforms from 0 to 800 ms after stimulus onset by averaging the EEG channel signals across 50 trials in the online experiment. Figure 2 shows the ERP waveforms of the “Fz,” “Cz,” and “Pz” electrodes for the three patients and two healthy controls. A highly similar P300 component elicited in all of the target curves.

**Figure 3:**
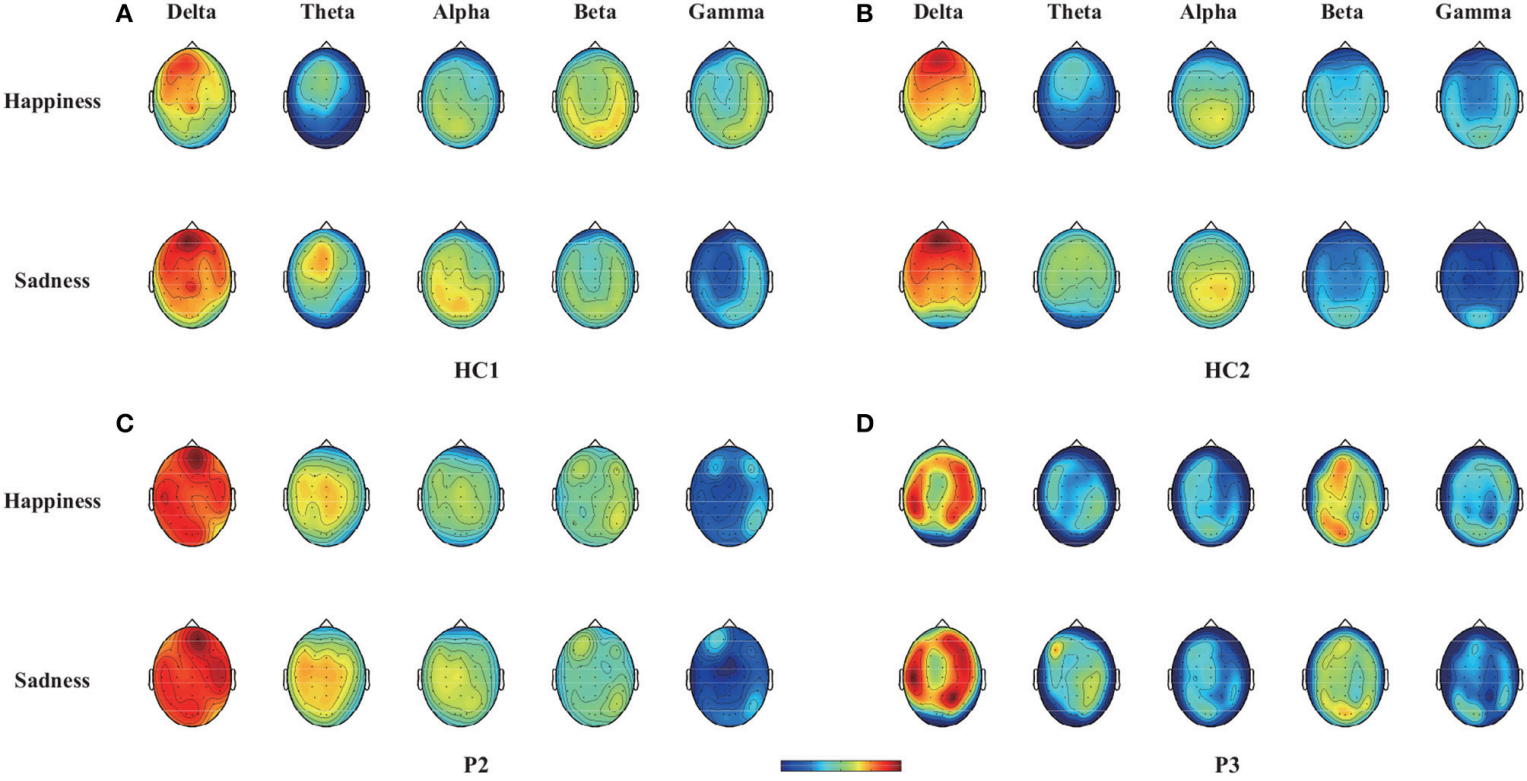
Topographical maps of the average DE features across trials with happy or sad emotional states in the five bands (delta, theta, alpha, beta, and gamma bands) for two healthy controls (HC1 and HC2) and two patients (P2 and P3). Note that the two healthy controls HC1 and HC2 and the two patients P2 and P3 achieved accuracies greater than 64% in offline spectral analysis. **(A)** Healthy subject HC1, **(B)** Healthy subject HC2, **(C)** Patient P2, **(D)** Patient P3.

To illustrate the influence of happy and sad movie clips on different brain regions, we projected the DE and CSP features onto the scalp to obtain the brain patterns of the five selected frequency bands across all trials with happy or sad movie clips. Here, brain patterns are considered neural activities in critical brain areas and frequency bands that share commonalities across trials during the happy and sad movie clips. Specifically, the DE features of each trial were calculated as the logarithmic power spectral density using the 18-s EEG signals of the target movie clip from each electrode. Figure 3 shows the topographical maps of the average DE features of the happy and sad emotions for the two healthy controls (HC1 and HC2) and the two patients (P2 and P3) who achieved accuracies higher than the significance level of 64% in spectral analysis. As shown in Figure 3, patients P2 and P3 had neural patterns that were similar to those of healthy controls HC1 and HC2: (1) In the delta band, the prefrontal area exhibited greater activation for sad emotions than happy emotions; (2) in the theta and alpha bands, the parietal area exhibited enhanced energy for sad emotions compared with happy emotions; and (3) in the beta and gamma bands, the lateral temporal and occipital areas exhibited greater activation for happy emotions than sad emotions.

**Figure 4:**
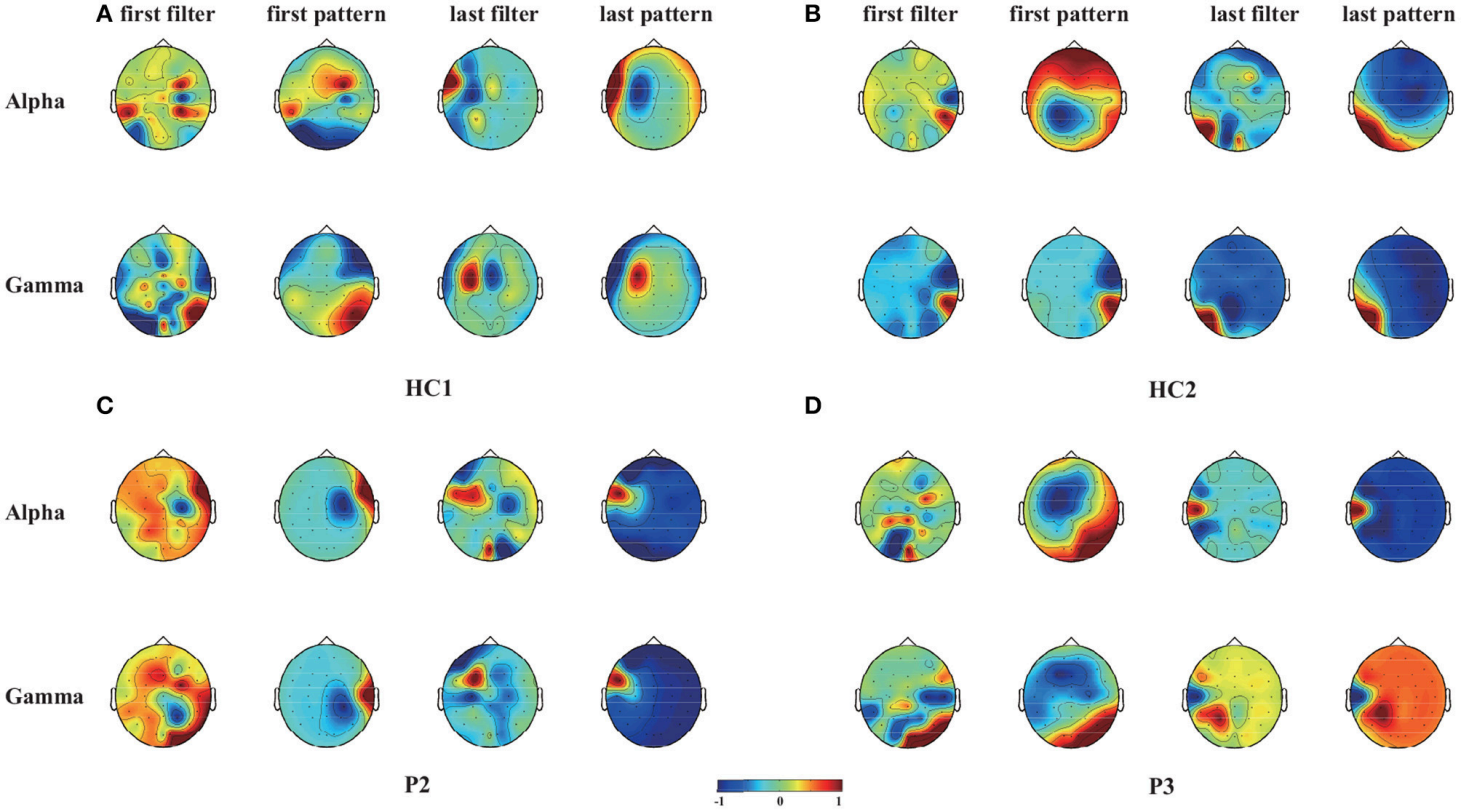
Scalp maps of two spatial filters (the first and the last rows of *W*) and the corresponding spatial patterns (the first and the last rows of *A*, where *A* = (*W*^−1^)^*T*^) for each of the alpha and gamma bands and for two healthy controls (HC1 and HC2) and two patients (P2 and P3). These CSP filters were trained using 50 trials gathered from the online evaluation. **(A)** Healthy subject HC1, **(B)** Healthy subject HC2, **(C)** Patient P2, **(D)** Patient P3.

Furthermore, the CSP filters were trained using the 50 trials obtained in the online experiment, which were used to project the original signals represented the weights of the original signals for achieving optimally discriminative projection (Yu et al., 2015). For two healthy controls (HC1 and HC2) and two patients (P2 and P3), we plotted the two spatial filters (the first and the last rows of **W**) and the corresponding spatial patterns (the first and the last rows of **A**, where **A** = (**W**^−1^)^*T*^ in Figure 4 as scalp maps for two frequency bands (alpha and gamma bands). The spatial filters/patterns are scaled by their maximum absolute values such that the values at each electrode position are normalized to the range of [-1,1]. Temporal and occipital asymmetries in the alpha and gamma bands can be observed from the CSP spatial patterns shown in Figure 4 for happy and sad emotion recognition. Specifically, the first filter/pattern was associated with more power over the right sensory cortical areas, because it was obtained by maximizing the variance of the first class (i.e., happy emotion). Accordingly, the last filter/pattern, corresponding to the second class (sad emotion), was associated with greater activation of the left sensory cortical areas. Such associations were clearly observed in both the healthy controls (HC1 and HC2) and the two patients (P2 and P3).

Among the three VS patients, one patient (P3) progressed to MCS 1 month after the experiment. More interestingly, the two patients (patients P2 and P3) with significant accuracies in both online P300 detection and offline spectral analysis improved their consciousness levels to a large degree 1 month after the experiment, while the other patients remained clinically unchanged. Specifically, their CRS-R scores improved from 9 and 5 (before the experiment) to 11 and 13 (1 month after the experiment), respectively. The CRS-R scores for each patient before and 1 month after the experiment were shown in Table 1.

## 4. Discussion

The detection of consciousness as well as residual cognitive function in patients with DOC is highly challenging but crucial for providing accurate diagnoses, selecting the optimal nursing strategies and ensuring overall quality of life. In this study, we used an EEG-based BCI to detect consciousness in patients with DOC. In this novel BCI paradigm, two movie chips, a crying movie clip and a laughing movie clip, were displayed on the left and right sides of the GUI, respectively, and flashed (appearing and disappearing) with a random interstimulus interval to evoke P300 potentials. When the movie clip was displayed, the corresponding audio clip was simultaneously played. Through the online P300 detection, the BCI system determined which movie clip the subjects attended to (i.e., responding to the instructions). Eight patients and eight healthy controls were involved in the experiment using our BCI system. They were instructed to focus on the crying or laughing movie clip and to count the flashes of the corresponding movie clip indicated by the instruction. Three of these patients (patients P2, P3, and P6) achieved online accuracies of greater than 64% (66–78%), which were considered significant. Furthermore, P300 responses (Figure 2) could be observed in these three patients. These three patients thus demonstrated the abilities to recognize emotion and follow commands.

It should be stressed that to perform the experimental tasks, many cognitive functions are required, such as language comprehension (i.e., understanding the task instructions), emotion processing (i.e., recognizing the emotional stimuli), and object selection (i.e., attending to the target movie clip). The absence of any of these cognitive functions could lead to failure of performing the task. Furthermore, negative results could not be used as evidence for a lack of consciousness, because false-negative findings in BCI studies are possible, even in healthy subjects. However, our positive results did indicate that such cognitive functions and residual consciousness existed in these patients.

Through the offline analysis, CSP and DE features in the delta, theta, alpha, beta, and gamma frequency bands were employed to classify the EEG signals recorded while the subjects watched the crying and laughing movie clips, corresponding to sad and happy emotions, respectively. Two patients (patients P2 and P3) achieved offline accuracies of the spectral analysis (66–68%) that were significantly greater than chance level. Furthermore, we projected the DE and CSP features of the happy and sad emotions onto the scalp in different frequency bands for two healthy controls (HC1 and HC2) and these two patients. To further illustrate the influence of happy and sad movie clips on different brain regions, we projected the DE and CSP features onto the scalp to obtain the brain patterns of the five selected frequency bands in Figures 3, 4. For or the sad emotion, the neural patterns had significantly higher delta responses at prefrontal sites and significantly higher theta and alpha responses at parietal sites. For the happy emotion, the neural patterns had stronger beta and gamma responses at the lateral temporal and occipital sites. Our results are partially consistent with the findings of previous works (Mühi et al., 2014; Zheng et al., 2016). For instance, studies (Onton and Makeig, 2009; Hadjidimitriou and Hadjileontiadis, 2012) have found that during positive emotion processing (e.g., emotion recognition of happy facial expressions), the energy of beta and gamma responses is enhanced. Increased delta band power was reported over parietal regions for negative stimuli compared with positive stimuli (Zheng et al., 2016). In addition, the experimental results in Figure 4 show that some of the asymmetric spatial patterns extracted by CSP were consistent with recent neurophysiological findings. For example, the event-related desynchronization in the alpha band (decreased alpha power) in response to stimulation is believed to represent increased sensory processing and, hence, has been associated with activation of task-relevant sensory cortical regions (Klimesch et al., 2007). Increases of gamma band activity have been observed over temporal regions in response to positive emotion, compared to negative emotion (Onton and Makeig, 2009). Taken together, stable brain patterns of the DE and CSP features associated with happy and sad emotions were observed in the two patients and the two healthy controls. Whether the happy and sad emotional responses were evoked using our BCI paradigm needs to be further confirmed in future studies.

For patients with brain damage, assessing emotion recognition ability is of substantial importance. First, emotion recognition is an important aspect of the cognition function of the human brain. Studies of the neural basis of emotion recognition (Adolphs, 2002) have suggested that somatosensory-related cortices in the right hemisphere play a critical role in emotion recognition. Areas of the amygdala, orbitofrontal and insular cortices are activated when subjects are engaged in emotion recognition (Adolphs, 2002). Second, basic emotional ability is relevant to the level of consciousness. Emotion and consciousness emerge as the result of neuronal activity in the brain (Damasio, 1999, 2003). Some studies have suggested that emotion is a possible facet of consciousness (Balconi and Lucchiari, 2007; Tsuchiya and Adolphs, 2007). Third, assessing emotion recognition ability may help us better understand other cognitive functions (e.g., language comprehension, working memory, and executive function) in patients with DOC. Several behavioral scales, including the FEAS, NEPSY-II, and MoCA, contain emotion recognition-based indices that are commonly used to evaluate the mental states of patients with schizophrenia, ADHD and Parkinson‘s diseases (Solomon et al., 2007; Marneweck and Hammond, 2014; Pitzianti et al., 2017). For patients with DOC, clinical assessment scales such as the CRS-R do not contain emotion recognition tasks. BCIs can provide both experimenters and patients with real-time feedback independent of motor responses, making detection and assessment of the emotion recognition abilities of patients with DOC possible, as demonstrated in this study. Using an EEG-based BCI, emotion recognition-related cognitive functions were successfully detected in 3 of 8 patients with DOC. Our results showed that the emotion recognition systems (including somatosensory related cortices in the right hemisphere) were at least partially effective for these three patients.

As previously discussed, misdiagnoses can occur based on behavioral observation scales such as the CRS-R. Therefore, BCIs can be used as a supportive bedside tool to assess patients residual cognitive ability. For instance, our experiment results showed that one VS patient (P3) was able to perform the BCI experimental task with an accuracy significantly higher than chance level. This result is consistent with previous fMRI (Monti et al., 2010) and EEG (Cruse et al., 2012b) data showing that some VS patients who are diagnosed based on the behavioral ceiteria might have residual cognitive function and even some level of consciousness. In fact, according to follow-up behavioral CRS-R assessments, this VS patient progressed to MCS 1 month after the experiment, thus supporting our BCI assessment result for this VS patient.

Notably, in our experiment, several patients achieved online accuracies (approximately 70%) that were significantly higher than chance level but much lower than the performance of the healthy subjects (which was generally higher than 90%). This discrepancy may be explained by two key factors. First, because the patients became easily fatigued, we could not collect sufficient training data before each online test block. Therefore, the performance of the classifier may have been affected by the insufficient amount of training data. Second, the patients with DOC had much lower levels of consciousness than the healthy subjects. Further studies are required to determine how to improve BCI accuracies for patients with DOC. A more elaborate preprocessing method could be employed to reduce the artifacts of the EEG signals, and the feature selection algorithms could be designed to adapt to these patients. Furthermore, our present offline results suggest that EEG signals recorded while the subjects watched the crying and laughing movie clips could be classified using emotion-related features. In future work, we may further integrate the P300 and emotion-related features to improve the ability to identify consciousness in patients with DOC.

## 5. Conclusion

In summary, BCIs can help patients with DOC who are very lack of motor responses to show emotion recognition. BCIs were thus verificated as an effective tool for the detection of related abilities. Given our focus on consciousness detection, we did not consider neutral stimuli in this study. Whether patients with DOC are able to differentiate their happy and sad emotions from neutral stimuli still remains unknown. This needs to be further confirmed in a future study.

## Author contributions

YL, JP, QX, and RY: designed this study; YL: designed the BCI systems, experiments and paradigms; JP: implemented the BCI systems; RY, QX, and YH: conducted the clinical assessments of participants; JP, HH, and YS: collected the BCI data; JP: analyzed the data; JP and YL: wrote the manuscript.

### Conflict of interest statement

The authors declare that the research was conducted in the absence of any commercial or financial relationships that could be construed as a potential conflict of interest.
